# Phylogenetic Signal Dissection of Heterogeneous 28S and 16S rRNA Genes in Spinicaudata (Branchiopoda, Diplostraca)

**DOI:** 10.3390/genes12111705

**Published:** 2021-10-27

**Authors:** Xiaoyan Sun, Jinhui Cheng

**Affiliations:** State Key Laboratory of Palaeobiology and Stratigraphy, Nanjing Institute of Geology and Palaeontology and Center for Excellence in Life and Palaeoenvironment, Chinese Academy of Sciences, No. 39, Beijing Eastroad, Nanjing 210008, China; xysun@nigpas.ac.cn

**Keywords:** concerted evolution, secondary structure, heterogeneity, Bayesian, spinicaudata

## Abstract

It is still a challenge to reconstruct the deep phylogenetic relationships within spinicaudatans, and there are several different competing hypotheses regarding the interrelationships among Eocyzicidae, Cyzicidae s. s., Leptestheriidae, and Limnadiidae of the Suborder Spinicaudata. In order to explore the source of the inconsistencies, we focus on the sequence variation and the structure model of two rRNA genes based on extensive taxa sampling. The comparative sequence analysis revealed heterogeneity across species and the existence of conserved motifs in all spinicaudatan species. The level of intraspecific heterogeneity differed among species, which suggested that some species might have undergone a relaxed concerted evolution with respect to the *28S rRNA* gene. The Bayesian analyses were performed on nuclear (*28S rRNA*, *EF1α*) and mitochondrial (*16S rRNA*, *COI*) genes. Further, we investigated compositional heterogeneity between lineages and assessed the potential for phylogenetic noise compared to signal in the combined data set. Reducing the non-phylogenetic signals and application of optimal rRNA model recovered a topology congruent with inference from the transcriptome data, whereby Limnadiidae was placed as a sister group to Leptestheriidae + Eocyzicidae with high support (topology I). Tests of alternative hypotheses provided implicit support for four competing topologies, and topology I was the best.

## 1. Introduction

Spinicaudata (Crustacea: Branchiopoda), the most diversified suborder of clam shrimps, is distributed globally (except in Antarctica) and occurs in a wide variety of terrestrial aquatic habitats, including temporary water bodies and inland saline pools and lakes [1,2,3,4]. This group includes around 200 extant species of 16 genera classified into 4 families: Eocyzicidae, Cyzicidae s. s., Leptestheriidae, and Limnadiidae [3,4]. The oldest confirmed spinicaudatans dated back to the Early Devonian, with 10 families occurring almost simultaneously, which suggested an early rapid radiation of the major spinicaudatan clades [5,6,7,8].

**Figure 1:**
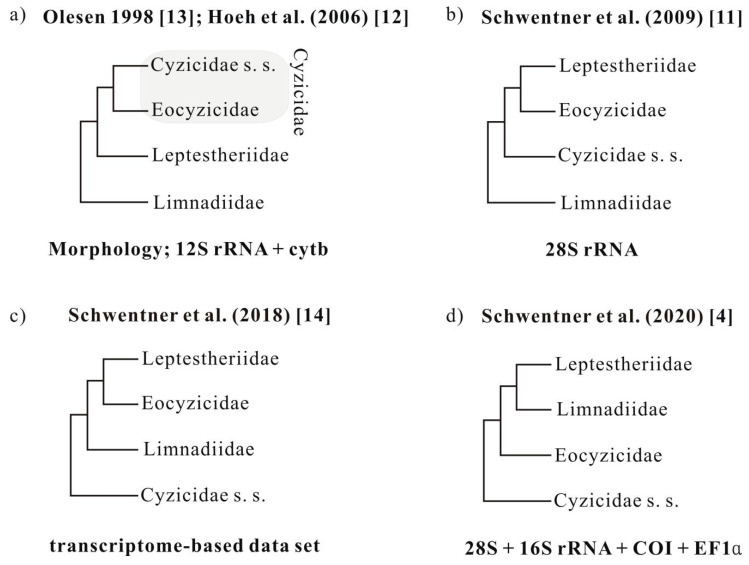
Four major competing hypotheses of relationships among extant spinicaudatan lineages derived from morphological or molecular data. (**a**): Olesen 1998 [13] and Hoeh et al. 2006 [12]; (**b**): Schwentner et al. 2009 [11]; (**c**): Schwentner et al. 2018 [14]; (**d**): Schwentner et al. 2020 [4].

Both morphological and molecular evidence strongly supported the monophyly of Spinicaudata [4,9,10,11]. However, the phylogenetic relationships and higher classification of Spinicaudata are still in a state of flux, as different data sets often support conflicting relationships (Figure 1). Early molecular phylogenies of spinicaudatans were inferred based on limited numbers of taxa and molecular loci. Hoeh et al. [12] recovered the monophyly of Cyzicidae s. s.+ Eocyzicidae, and Limnadiidae as a sister group to the ((Cyzicidae s. s.+ Eocyzicidae) + Leptestheriidae) clade, based on the combined *12S rRNA* and *cytb* mtDNA data set. This is congruent with a cladistic analysis based on morphological data by Olesen [13] (Figure 1a). Bayesian and maximum likelihood analyses inferred from *28S rDNA* segments revealed that *Leptestheria* and *Eocyzicus* were sister groups (Figure 1b) [11,12]. Schwentner et al. [14] proposed the first transcriptome phylogeny and provided robust support for the deep relationships among Spinicaudata, where the Eocyzicidae + Leptestheriidae clade was a sister group to Limnadiidae, and Cyzicidae s. s. was recovered at the base of Spinicaudata (Figure 1c). This is contrasted with the morphology-based hypothesis, which suggests Cyzicidae s. s. is the closest to Leptestheriidae. The most species-rich phylogenetic analyses of Spinicaudata by Schwentner et al. [4] using four molecular loci (i.e., cytochrome *c* oxidase subunit I of mitochondrial DNA (*COI*), elongation factor 1-α (*EF1α*) gene, *16S* and *28S rRNA* genes (Figure 1d)) identified a clade comprising Limnadiidae and Leptestheriidae as a sister group to Eocyzicidae. Thus, many outstanding questions remain unresolved due to apparent conflicting phylogenetic signals in molecular data sets, although our understanding of the phylogenetic relationships among families of Spinicaudata has been improved. Moreover, the phylogenetic relationship within Leptestheriidae was not well resolved [4]. The placement of *Imnadia* and *Limnadia* of Limnadiidae has also been inconsistent and unresolved [4,11,12,15,16].

Non-phylogenetic signal has multiple and disparate sources [17,18]. In practice, it mainly comes from the incorrect identification of orthologous genes, compositional heterogeneity, the presence of fast evolving taxa, the incorrect reconstruction of multiple substitutions occurring at a given position, or the use of suboptimal models of evolution [18]. These effects may result in incongruent, yet statistically highly supported, phylogenetic trees. For example, most models of evolutionary change assume that base composition is stationary through time and across lineages. This assumption may be violated by many data sets where compositional heterogeneity or a combination of compositional heterogeneity and rate heterogeneity of sequences between taxa may compromise phylogenetic inference [19]. When sequences in a multiple alignment have undergone so many multiple substitutions that apparent distances largely underestimate the real genetic distances, the alignment is said to be saturated [18]. This process causes homoplasy in nucleotide gene data. Thus, substitutional saturation is one of the most frequently discussed causes of phylogenetic artifacts. Ribosomal RNA (rRNA) is the central component of ribosomes. The rRNA genes are comprised of several highly conserved regions interleaved with variable regions, and are conserved in both linear sequences and secondary structures, making them most commonly used for phylogenetic studies. For spinicaudatans, molecular phylogenies have been mostly based on rRNA genes. It is now widely recognized that the multiple copies of ribosomal DNA (rDNA) in the genome are homogenized by different mechanisms, collectively termed concerted evolution [20]. Nevertheless, high levels of intragenomic and intraspecific polymorphism have also been reported for several eukaryotes [21,22,23]. These suggested that ribosomal genes have been subjected to a mixed process of concerted and birth-and-death evolution [24]. Ribosomal RNA loop and stem regions are under different selective constraints. Compared to loop regions, the base-paired double helical regions (stem regions) in rRNA genes evolve under high functional constraints in the form of compensatory mutations, to maintain energetically stable secondary structures [24,25]. The application of specific models in tree reconstructions is therefore hampered by sequence heterogeneity, covariation patterns of paired sites, and excessive homoplasy in some loop regions. Letsch and Kjer [26] explored the potential and pitfalls of modelling ribosomal RNA data in phylogenetic tree reconstruction, and suggested that RNA models often failed to recover reasonable trees when single-stranded regions were excessively homoplastic, because these regions contributed a greater proportion of the data when covarying sites were essentially down-weighted. However, recent developments in phylogenetic methodology also indicated that the non-phylogenetic signals could be reduced by improving the quality of primary alignments, minimizing compositional heterogeneity, detecting multiple substitutions, and using the most realistic model of sequence evolution [18].

Resolving phylogenetic relationships in a very deep divergence poses a major challenge for phylogenetic analysis even when using very long gene sequences [27]. Previous molecular studies have highlighted the difficulty in inferring deep phylogeny of Spinicaudata [4,9,10,11,12,14]. Thus, it would be significant to proactively dissect the potentials for phylogenetic noise and signal in a candidate data set. A recent public molecular data set with extensive taxon-sampling created by Schwentner et al. [4] provided an opportunity to test these hypotheses. The present study aims to get a better understanding of phylogenetic relationships within the Suborder Spinicaudata. We focused on the sequence variation of *28S* and *16S rRNA* genes in Spinicaudata at different levels, in order to improve the quality of alignments. Based on the refined structure model and structurally aided sequence alignments, we further conducted phylogenentic reconstruction with the doublet models, which assigned an RNA substitution matrix to base pairs. Then, we provided evidence that non-phylogenetic signal could mislead phylogenetic inferences of Spinicaudata. Finally, we employed statistical tests of significance to test (1) hypotheses of spinicaudatan relationships, (2) the phylogenetic relationships within Leptestheriidae, and (3) the internal relationships of the limnadiid genera.

## 2. Materials and Methods

### 2.1. Taxon Sampling

We sampled data from GenBank (http://www.ncbi.nlm.nih.gov accessed on 5 October 2020) in a way that maximized the taxonomic sampling among spinicaudatans, following the published molecular data set of Spinicaudata [4], combined with 8 newly generated sequences, resulting in a data set (Table S1). The data set is composed of 102 ingroup species (including 50 unspecified species), representing 14 of the 16 described spinicaudatan genera. Fifteen species from the orders Notostraca and Anostraca and the Suborder Laevicaudata were used as outgroups. In total, we included *28S rRNA* sequences from 262 specimens and *16S rRNA* sequences from 116 specimens, representing all major lineages within spinicaudatans. Samples generating new sequences were collected from Chengde of Hebei Province, and Changchun of Jilin Province, China. Voucher specimens were stored in 96% ethanol and deposited in Nanjing Institute of Geology and Palaeontology, Chinese Academy of Sciences.

### 2.2. DNA Extraction, PCR Amplification and Sequencing

Genomic DNA was extracted from a single specimen with DNeasy tissue kit (Qiagen, Hilden, Germany) following the directions of the manufacturers. The primer pairs *16S*A/*16S*B [28] and LCO1490/HCO2198 [29] were used to PCR amplify a 512-base pair (bp) fragment of *16S* and a 512-bp fragment of *COI*, respectively. Primer pair *28S*F/*28S*R [30] was used to obtain sequences from *28S* (1022 bp). For the amplification of 769 bp of *EF1α*, the primer pair HaF2For1/2R53ST [10] was used. The PCR amplifications followed Schwentner et al. [11]. The PCR products were gel purified using the QIAquick Gel Extraction Kit (Qiagen) and then sequenced in both directions with PCR primers by Sangon Biotech Co. (Shanghai, China). All sequences were submitted to GenBank (accession numbers MZ313265-MZ313268 and MZ318668-MZ318671; Table S1).

### 2.3. Sequence Alignment and Secondary Structure Prediction

Based on the published secondary structure maps of Spinicaudata [31], the D1–D2 region of *28S rRNA* was aligned using Clustal X [32] in the first step. The partial *16S rRNA* structure (the portion corresponding to domains IV and V) was derived from the proposed reference secondary structure for branchiopods [33,34]. Then, putative helices were distinguished by searching conserved motifs located in the uninterrupted base pairing regions. Less-conserved regions were folded using RNAstructure 5.2 [35]. The identified stem and loop regions were used for partitioning analysis and the pairing nucleotide positions were applied to the doublet model algorithm [36] implemented in MrBayes 3.2 [37]. The amino acid sequences of *EF1α* and *COI* were aligned using MUSCLE implemented in MEGA 7.0 [38]. The corresponding nucleotide sequences of *EF1α* and *COI* were then aligned using the aligned amino acid sequences implemented in DAMBE 6 [39].

### 2.4. Phylogenetic Analyses

Phylogenetic reconstructions were conducted using Bayesian methods on *28S rRNA*, *16S rRNA* and concatenated sequences (*28S* + *16S rRNA*, *28S* + *EF1α* and *28S* + *16S rRNA* + *COI* + *EF1α*). We identified the best-fitting nucleotide substitution models using MrModeltest version 2.2 [40]. According to the method proposed by Brandley et al. [41], which preferred a more partitioned strategy, the concatenated rRNA data set was split into six partitions: *16S* loops, *16S* stems, *28S*V1 loops, *28S*V1 stems, *28S*V2 loops, and *28S*V2 stems. We estimated saturation for five sorts of subsets of the concatenated data set: the stem sites of rRNA genes, the loop sites of rRNA genes, the first codon positions, the second codon positions, and the third codon positions of protein-coding genes. Considering that the third codon positions have experienced substantial substitution saturation and therefore were excluded from further phylogenetic analyses, Bayesian analyses were run with 2 simultaneous runs of 4 chains each (heating T = 0.1), random starting trees and trees sampled every 1000 generations for 1.0 × 10^7^ generations. Convergence was checked using the overlay log-likelihood (lnL) scores plot of 2 independent runs, standard deviation of split frequencies (<0.01 threshold) and potential scale reduction factor (PSRF, close to 1.0) for the model parameters and clade supports. Burn-in phase was set at 25% of the results. Bayesian posterior probability (BPP) was calculated during the stationary phase.

### 2.5. Non-Phylogenetic Signal Dissection

#### 2.5.1. Network Analysis

In order to visualize the signals and conflicts presented in the concatenated data set (*28S* + *16S rRNA* + *COI* + *EF1α*), we used SplitTrees 4.11.3 [42] to calculate phylogenetic networks based on the neighbour-net algorithm. Both logDet (corrects for bias in base composition) and p-distance transformations were evaluated.

#### 2.5.2. Evaluation of Compositional Heterogeneity and Substitutional Saturation

To test the base compositions and substitutional saturation of stem and loop regions of rRNA genes, all alignments were divided into unpaired (loop) and paired (stem) partitions according to a consensus secondary structure. To visualize the sequences’ heterogeneity, the GC percentage score and branch length of each genus generated by DAMBE 6 [39] was plotted. To measure whether the taxa in our data sets are compositionally heterogeneous, we conducted posterior predictive analysis (PPA) under the GTR + G model, as implemented in PhyloBayesv4.1c [43]. We excluded the taxa with the deviating nucleotide composition to reduce compositional heterogeneity in the data sets. The level of nucleotide substitution saturation was assessed using DAMBE 6 [39], which estimates an “index of substitution saturation (Iss)”, based on the notion of entropy in information theory.

### 2.6. Testing Support for Competing Hypotheses

To statistically test the validity of several key phylogenetic relationships within spinicaudatans, approximately unbiased (AU) test [44], the Kishino–Hasegawa (KH) test [45], and the Shimodaira–Hasegawa (SH) test [46] analyses were performed on the concatenated sequences (*28S rRNA* + *16S rRNA* + *EF1α* + *COI*). Alternative hypotheses included: (1) the highest likelihood topologies with the higher relationships of Spinicaudata, calculated from the concatenated sequences of *28S* + *16S rRNA* + *COI* + *EF1α*, (2) the topology with the highest likelihood estimated with the relationships within Leptestheriidae, and (3) the internal relationships of the limnadiid genera. Site-wise loglikelihoods for all trees were estimated using Tree-Puzzle [47] and used as input for CONSEL 0.1 [48]. Multiscale bootstrap resampling was conducted with ten sets of 10,000 replicates each, with scale parameters ranging from 0.5 to 1.4.

## 3. Results

### 3.1. Variation and Conserved Motifs in the 28S rRNA and 16S rRNA Secondary Structure among Spinicaudata Lineages

**Figure 2:**
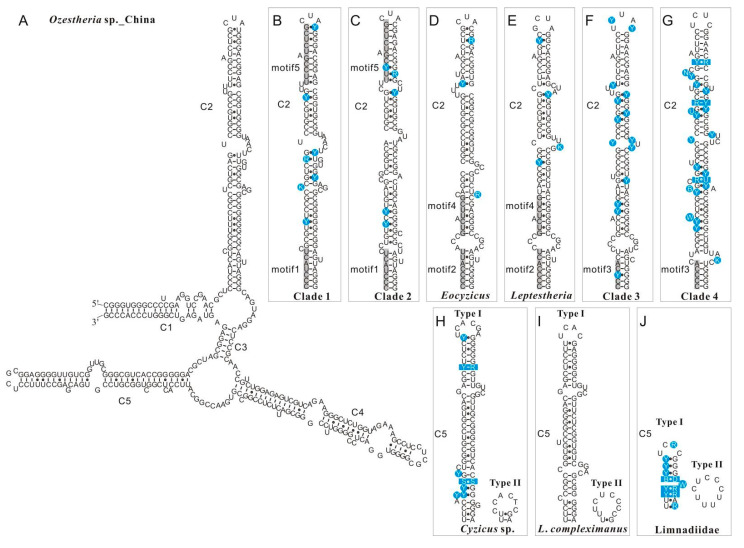
Secondary structure and helix numbering of *28S rRNA* D2 expansion fragment (V2). (**A**). Secondary structure diagram for *28S rRNA* V2 of *Ozestheria* sp. from China. (**B**–**G**). Variations on the V2-C2 helix across main clades of Spinicaudata (the conserved motifs are indicated in grey). (**H**–**J**). Polymorphism on the V2-C5 helix of *Cyzicus* sp. (WRH-2009), *Leptestheria compleximanus* and of Limnadiidae. The variable sites are marked in blue circles. Clade 1: *Ozestheria*; clade 2: *Cyzicus*; clade 3: *Paralimnadia* + *Australimnadia* + *Limnadopsis*; clade 4: *Metalimnadia* + *Eulimnadia* + *Gondwanalimnadia* + *Calalimnadia*.

Predicted secondary structures of both *28S rRNA* and *16S rRNA* were compared among all spinicaudatan species to further investigate the sequence variation. The *28S rRNA* sequence fragments for comparative structure analysis ranged from 378 bp to 521 bp in length. It corresponded to D1 variable region (V1) and D2 variable region (V2), and the alignment had 614 positions. The length variation of the *28S* V1 segment within spinicaudatan species was minimal (only 2 bp difference). The *28S* V1 segment comprised four main compound helices labelled B13_1, B14, B16 and B17 across all spinicaudatan species, which were highly conserved in the *28S* core structure of branchiopods. All predicted secondary structures of *28S rRNA* V2 consisted of five compound helices (C1–C5), which was similar to those of *Cladocera* [31]. Secondary structure diagrams for *28S rRNA* V2 of *Ozestheria* sp. (China) are shown in Figure 2A. Compound helix C2 in the V2 region was highly conserved in spinicaudatans. A gallery of structures representing the compound helix C2 motifs is presented in Figure 2B–G. Base changes were mostly observed on the stem instead of the loop regions. The structural alignment revealed five conserved motifs in C2 that could provide supports for major phylogenetic lineages of Spinicaudata: (1) a conserved seven homologous base-pairing motif (5′-CCCUACU-3′, motif1, Figure 2B,C) was observed in clade 1 (*Ozestheria + Caenestheriella + Caenestheria*) and clade 2 (*Cyzicus*). (2) Another 5′-CCCCAYU-3′ motif (motif2, Figure 2D,E) occurred in *Eocyzicus* and *Leptestheria*. (3) 5′-CCYCA-3′ (motif3, Figure 2F,G) occurred in Limnadiidae. (4) Motif4 (5′-GUCUYYG-3′, Figure 2D,E) occurred in the second helix of C2 for *Eocyzicus* and *Leptestheria*. (5) Motif5 (5′-UUYCGUCUCG-3′, Figure 2B,C) occurred in the terminal helix of C2 for clade 1 and clade 2.

Helix C3 is highly conserved in the higher eukaryotes, and is the most basal helix to several compound helices [49,50]. Helix C3 is six base-pairs long in the spinicaudatans. The comparative sequence analysis revealed that the V2-C5 exhibited extensive length polymorphisms in spinicaudatans, with length ranging from 9 to 82 bp. This length variation was found in different populations within a single species (e.g., *Eulimnadia cylindrora* and *Leptestheria compleximanus*). Due to the length variations, two secondary structure types including a long C5 helix (named type I), and a reduced type II were identified among spinicaudatans, for example, two types in *Eocyzicus* (Figure 2H) and *Leptestheria compleximanus* (Figure 2I). This polymorphism was also generally observed in the Family Limnadiidae (Figure 2J). Excluding the V2-C5 region, the two genes of a single species had a 100% sequence similarity.

The sequences of *16S* rRNA analyzed in this study ranged from 460 to 570 bp, and the alignment had 627 positions. It corresponds to the part of domains IV and V of *16S rRNA*, including stems E24, E25, E26, E27, E28, F1, G2, G3, G6, G7, G9, G15 and G16. Stems E22, E23, E21, E18, E1, G1, G16 and G17 did not form due to the small size of the fragment analyzed. The secondary structure of the part of domains IV–V predicted for spinicaudatans was similar to that of *Triops granarius* (Notostraca: Triopsidae) [34]. The structure and motifs were highly conserved, in which E22, E25, E26, E27, E28, G2, G6 and G7 had more than 75% sequence similarity respectively. For example, helix E25 of domain IV was typically initiated by three highly conserved couplets (composed of Gs and Cs) followed by bilateral bulges. The terminal couplets of helix E25 (A·U and G·C interactions) were highly conserved among all spinicaudatans, whereas the loop terminating this helix was highly variable. Helix E26 was lengthened to include the motif: CGCGGU, and was terminated by a hairpin loop of 6 bp in length among all spinicaudatans; helix E28 was extended to include the conserved GUCUCY motif. Helix E27 was adjusted to the shortest helices, which only contained three pairing bases, but adjacent part on both sides of the helix, the pivotal motifs: “CGAA” or “A” were present, which helps to recognize this hairpin. Helices E26, E27 and E28 were highly conserved in length and primary sequence in almost all spinicaudatans. Similarly, significant heterogeneity was observed in the domain IV and V of the *16S rRNA* gene of *Cyzicus setosus*, *Ozestheria lutraria*, *Leptestheria kawachiensis*, and *L. dahalacensis*. For example, two different types of secondary structure of the *16S rRNA* gene were detected within a single species, *Ozestheria lutraria*, which contained 54-bp deletion (DQ470603). The 54-bp deletion occurred at helix G3 of domain V. Excluding the G3 deleted region, the two types had a 100% sequence similarity. Relative to other *16S* rRNA sequences of *O. lutraria*, *O. lutraria* (DQ470603) showed higher GC-content (39% vs. 37%). This phenomenon was also observed in *Leptestheria kawachiensis* and *L. dahalacensis*. Helix G9 was absent from *Eocyzicus mongolianus*, *E. orientalis* and *Limnadia* sp. (JRdW-2005). Another interesting feature was a long deletion (105 bp) in *Cyzicus setosus* (DQ310668), occurring at helices E18 and E1 of domain IV, and helices G3, G7 and G9 of domain V. Excluding the 105 bp deleted region, the two types of *Cyzicus setosus* had a 95.9% sequence similarity. Helices E18, G7 and G9 were also absent from *Limnadia* sp. (JRdW-2005). In *Eocyzicus mongolianus* and *E. orientalis*, helix E18 was reduced, consisting of just 5 paired bases, relative to other eocyzicids, 8 paired bases.

### 3.2. Data Exploration: Compositional Heterogeneity, Relative Homoplasy and Supernetwork

**Figure 3:**
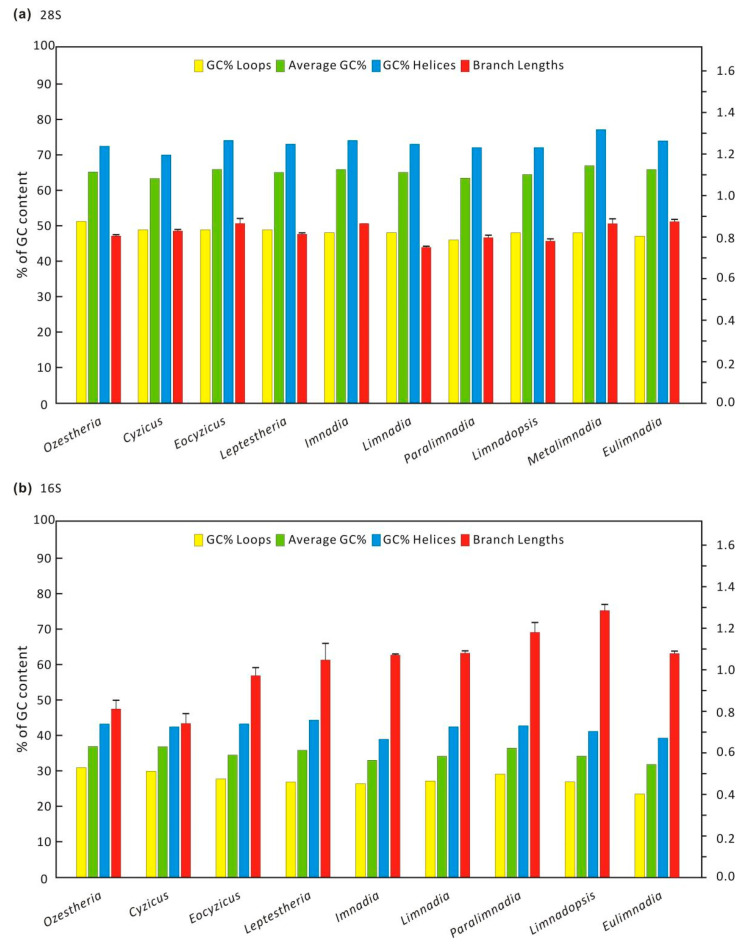
Mean values of base composition and average branch lengths (brls) with standard deviation (σ) values of each clade. Paired sites show a much higher GC% content compared to unpaired sites. (**a**): *28S* *rRNA*; (**b**): *16S* *rRNA*.

Among the 262 spinicaudatan specimens sampled for *28S rRNA*, the GC content ranged from 60% to 68%, and *Ozestheria*, *Eocyzicus*, *Imnadia*, *Metalimnadia* and *Eulimnadia* (Figure 3) had a high GC content (>65%). The GC content in the unpaired regions was approximately 50%, whereas paired sites had a much higher GC content compared to unpaired sites (69–77%). This pattern was also identified by the observation of entire large-subunit rRNA in all three domains of life [51]. Posterior predictive analysis of homogeneity composition showed that none of the species in *28S rRNA* data set were compositionally heterogeneous (Table S2). A comparison of branch lengths (Figure 3) revealed a high degree of similarity between the branch lengths, and the distribution of branch length was not biased towards any high values. Among the 116 spinicaudatan specimens sampled for *16S rRNA*, the GC content ranged from 32% to 37%. *Ozestheria* had the highest overall GC content (37%) and *Eulimnadia* had the lowest (32%). Similarly, paired sites of *16S rRNA* had a much higher GC content (39–44%) compared to the unpaired sites (24–30%). The nucleotide composition difference between the unpaired and the paired regions was associated with the expectation of functional constraints which might arise naturally from the process of RNA folding on the natural rRNA molecules [52].

Posterior predictive analysis of homogeneity composition showed that seven of the species in *16S rRNA* data set were compositionally heterogeneous. As displayed in Table S2, the among-lineage compositional heterogeneity of the *16S rRNA* data set was reduced by removing the compositionally heterogeneous taxa with high Z scores (Z-scores > 2 *p* < 0.05). A comparison of branch lengths (Figure 3) indicated that *Limnadopsis* exhibited the longest branch length (1.285), while *Cyzicus* had the shortest branch length (0.741). The difference was not significant.

The substitutional saturation of stem and loop regions of each rRNA gene was estimated within spinicaudatan genera, and a summary of saturation tests, including other alignment parameters, is given in Table 1. The saturation tests revealed significant differences between the paired and the unpaired positions. In the loop portions of all data sets, the observed ISS was always larger than the critical value for the index of substitution saturation (I_SS.c_), except *28S* *rRNA* of *Limnadopsis*, which indicated excessive homoplasy and a loss of phylogenetic information in the unpaired positions. In contrast, most of the stem portions of rRNA data sets were free of saturation, which suggested that stem positions in current data sets contain more reliable phylogenetic signals, compared to loop regions. The saturation test results of the loop and stem partitions were also compared to the saturation test result of the combined data set of Spinicaudata. As displayed in Table 1, saturation vanished in the combined data set of *28S* + *16S rRNA* + *COI* + *EF1α*.

**Figure 4:**
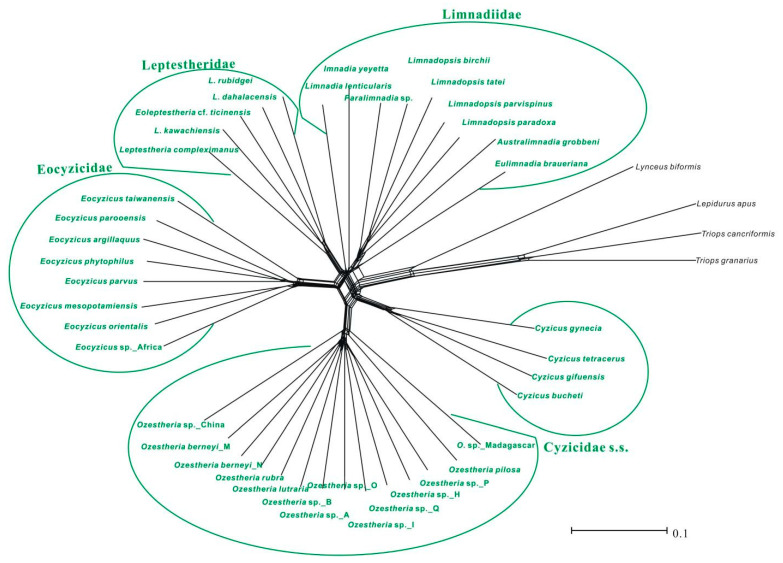
The split-tree phylogenetic networks based on logDet distance transformations of the concatenated data set (*28S* + *16S rRNA* + *COI* + *EF1α*). Members of Spinicaudata are scaled in green curves; the scale bar represents the split support for the edges.

Phylogenetic networks based on logDet and p-distance transformations had very similar results, hence we only present those based on logDet distances (Figure 4). The network graph gave a first indication of signal-like patterns and conflict present in the concatenated data set. The strongest split separated outgroups from Spinicaudata, and within Spinicaudata, each major group was always resolved as monophyletic. As displayed in Figure 4, the central part of the graph is dominated by many contradicting edges, which represents incompatible splits in the data set.

### 3.3. Phylogenetic Relationships among Main Clades of Spinicaudata

#### 3.3.1. A *28S rRNA*-Based Phylogeny of Spinicaudata

**Figure 5:**
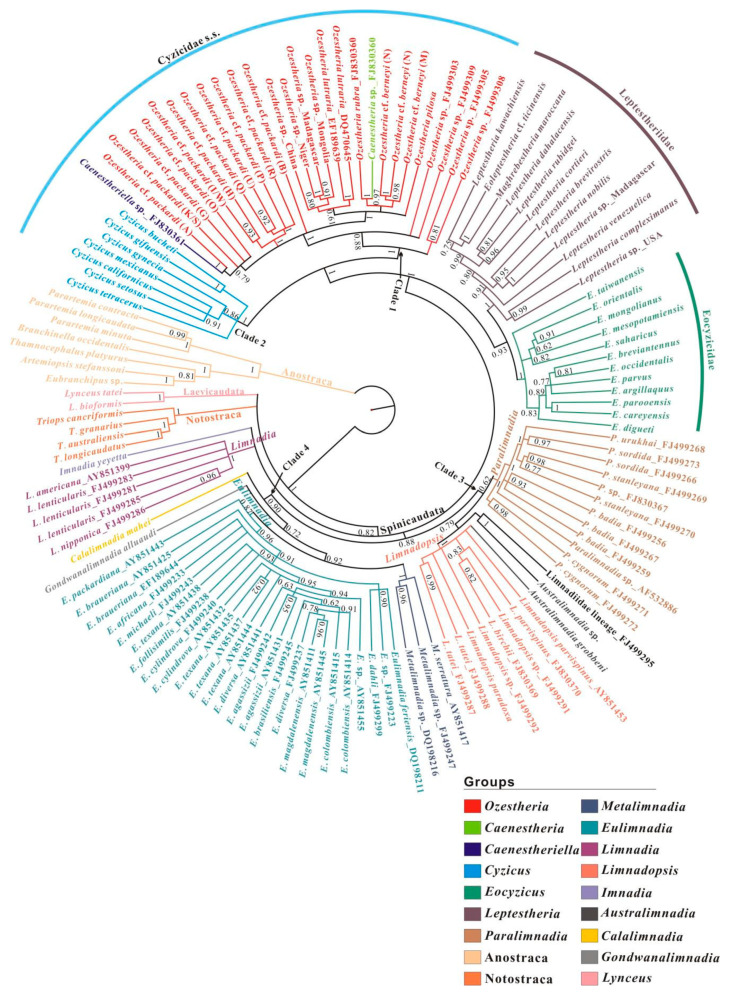
The 50% majority consensus BI tree showing the branching pattern among different species and populations of spinicaudatans (color-coded) based on *28S rRNA* gene, using a 16-state doublet model. Bayesian posterior probability (PP) support values are reported on the nodes. Clades 1 to 4 are the same as in Figure 2.

A Bayesian phylogenetic analysis of *28S rRNA* resulted in four major monophyletic groups (Figure 5): (1) Cyzicidae s. s. (BPP = 1.0), (2) Leptestheriidae (BPP = 1.0), (3) Eocyzicidae (BPP = 1.0), and (4) Limnadiidae (BPP = 0.82). Cyzicidae s. s. split into *Ozestheria* (clade 1) and *Cyzicus* (clade 2); clade 1 consisted of 26 *Ozestheria* species, 1 unclassified *Caenestheriella* species and 1 unclassified *Caenestheria* species. Clade 2 comprised 7 species of Holarctic *Cyzicus*. Eocyzicidae and Leptestheriidae were sister groups (BPP = 0.93), which was congruent with the conclusion of Schwentner et al. [11]. A monophyletic group consisting of Cyzicidae s. s., Eocyzicidae, and Leptestheriidae was also supported with high support value (BPP = 1.0). Limnadiidae splitted into four clades: *Imnadia*, *Limnadia*, (*Calalimnadia* + (*Gondwanalimnadia* + (*Eulimnadia* + *Metalimnadia*))) (clade 3), and ((*Limnadopsis* + *Australimnadia* + two unclassified Limnadiidae lineage) + *Paralimnadia*) (Clade 4). The branching pattern within clade 3 is consistent with the conclusion of Bellec and Rabet [16]. In clade 4, *Eulimnadia* species from six geographic regions constituted a monophyletic group as a sister to *Metalimnadia*. *Gondwanalimnadia* and *Eulimnadia* + *Metalimnadia* formed a monophyletic group. Hermaphroditic *Calalimnadia* was the basal group of clade 4. This result is also in congruence with the conclusion of Bellec and Rabet [16]. The sister group relationships between *Imnadia*, *Limnadia*, and clade 3 or clade 4 have not been resolved.

Additionally, the *28S* D2 sequences of *Caenestheriella* sp. (FJ830361) and *Ozestheria* cf. *packardi* (lineage A) sensu Schwentner et al. [53] differed by 1 bp, and they might represent the same species. The *28S* D2 sequences of *Caenestheria* sp. (FJ830360) and *O*. *rubra* differed by 2 bp, and they also belonged to the same species. Thus, the monophyly of genus *Ozestheria* was supported with high support value (BPP = 1.0). For the Leptestheriidae, the 553-bp sequence of *Eoleptestheria* cf. *ticinensis* (MN585030, Australia) corresponded to part of D2 and D3 regions of *28S rRNA*, and stems C1 and C2 of D2 region did not form; the sequences of *28S* D2–D3 regions of *Eoleptestheria ticinensis* (MN585093, Italy) and *Eoleptestheria* cf. *ticinensis* (MN585030, Australia) had a 98.6% sequence similarity; the sequences of *28S* D2 region of *Eoleptestheria ticinensis* (MN585093, Italy) and *Eoleptestheria ticinensis* (MN585030, Australia) differed by only 5 bp (2 bp in stem positions and 3 bp in loop positions). Sequence of *Eoleptestheria* cf. *ticinensis* (MN585030, Australia) was consequently excluded from phylogenetic analyses. A monophyletic group consisting of *Maghrebestheria* and *Eoleptestheria ticinensis* was recovered (BPP = 1.0). A sister group relationship between *Maghrebestheria* + *Eoleptestheria* and *Leptestheria kawachiensis* was supported (BPP = 0.75).

#### 3.3.2. A *16S rRNA*-Based Phylogeny of Spinicaudata

The Bayesian analysis of *16S rRNA* fragment was similar to the analysis of *28S rRNA* data set in recovering 2 of 4 major monophyletic groups: Leptestheriidae (BPP = 1.0) and Eocyzicidae (BPP = 1.0), while it was not the same with Limnadiidae (Figure S1). *Cyzicus* and *Ozestheria* were monophyletic, but the phylogenetic positions were unresolved. *Imnadia* and *Limnadia* formed a monophyletic clade which was a sister to (Leptestheriidae + all other limnadiids) (BPP = 0.78). The most striking differences compared to the *28S rRNA* analysis were: (1) The most species-rich clade of Leptestheriidae had a comb-like topology; (2) Leptestheriidae was nested within paraphyletic Limnadiidae; (3) Eocyzicidae was a sister group to ((*Imnadia* + *Limnadia*) + (Leptestheriidae + all other limnadiidae)) (BPP = 1.0).

#### 3.3.3. Combined *28S* + *16S rRNA* Analysis

**Figure 6:**
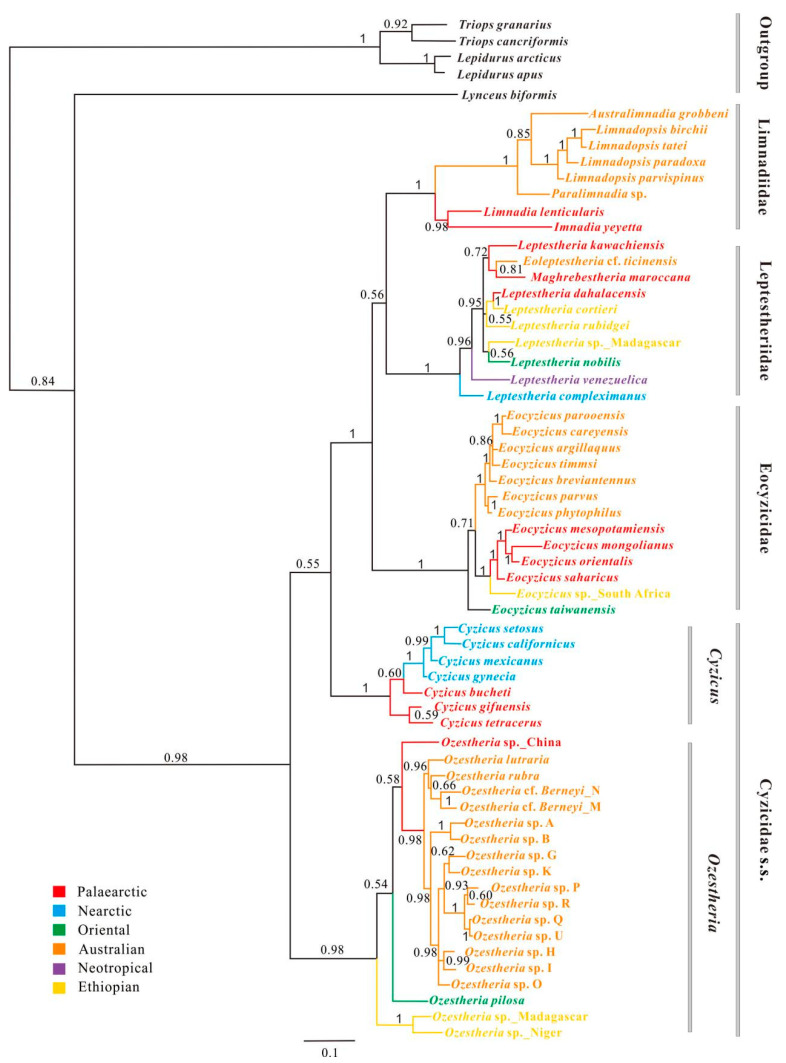
Bayesian inference majority rule tree based on a combined analysis of *28S rRNA* and *16S rRNA*, using a 16-state doublet model. Bayesian posterior probability (PP) support values are reported on the nodes.

The Bayesian combined rRNA analysis with structural partitions recovered 3 of 4 major monophyletic groups: Leptestheriidae (BPP = 1.0), Eocyzicidae (BPP = 1.0) and Limnadiidae (BPP = 1.0), while Cyzicidae s. s. was paraphyletic (Figure 6). The Bayesian-combined rRNA analysis supported a sister group relationship between Leptestheriidae and Limnadiidae, but the support value for this sister relationship was low (BPP = 0.56). Eocyzicidae was placed as a sister group to Leptestheriidae + Limnadiidae (BPP = 1.0). This branching pattern has been consistently and strongly supported by a previous study [4] based on combined data analyses of *COI*, *16S rRNA*, *EF1α* and *28S rRNA*. Paraphyletic Cyzicidae s. s. was divided into two well-supported genera, *Ozestheria* and *Cyzicus*. The support value for *Cyzicus* as a sister group to ((Leptestheriidae + Limnadiidae) + Eocyzicidae) was low (BPP = 0.55). For the Limnadiidae, it was well supported (BPP = 0.98) that *Imnadia* and *Limnadia* formed a monophyletic clade (BPP = 1.0) as a sister group to the Australian ((*Limnadopsis* + *Australimnadia*) + *Paralimnadia*) clade. For the Leptestheriidae, the branching pattern of ((*Maghrebestheria* + *Eoleptestheria*) + *Leptestheria kawachiensis*), as recovered in the analysis of *28S rRNA* data set, was supported with an overall reduction in clade support (BPP = 0.72–0.81); *Leptestheria compleximanus*, being the earliest leptestheriid lineage, received significant support (BPP = 0.99).

#### 3.3.4. *28S rRNA* + *EF1α* Combined Analysis

**Figure 7:**
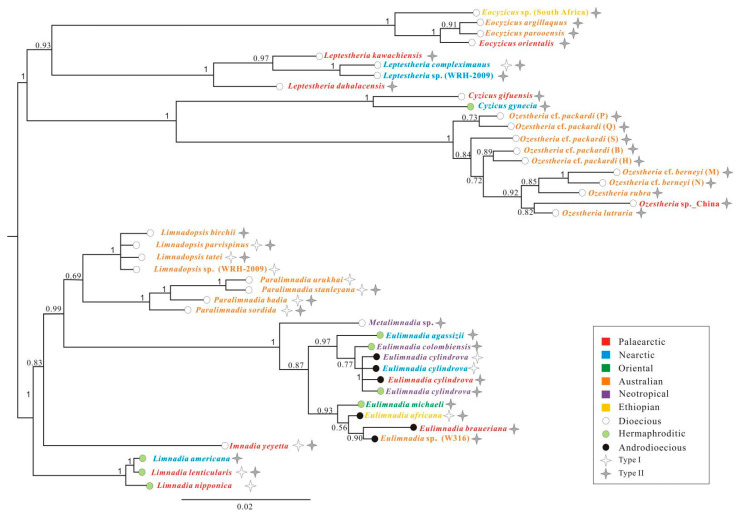
Different base-pairing types of *28S rRNA* V2-C5 and reproductive modes placed on the Bayesian phylogenetic tree of Spinicaudata. The consensus tree was constructed with Bayesian inference, based on *EF1α* + *28S rRNA*. Bayesian posterior probability (PP) support values (>0.5) are shown in the tree.

In order to investigate the association between rDNA polymorphism and the mode of reproduction, we conducted a phylogenetic analysis based on combined *28S* *rRNA* and *EF1α* genes. Breeding system determinations for clam shrimp populations were identified in recent studies [15,54,55,56,57,58,59,60,61]. We chose the 42-taxa Bayesian tree to represent the correlation of rDNA polymorphism with reproductive modes. An analysis of the combined *28S* + *EF1α* genes resulted in a similar topology (Figure 7) for main spinicaudatan clades to that of the *28S rRNA* data set alone. The difference compared to the *28S rRNA* analysis was the branching pattern of Limnadiidae. Within the Family Limnadiidae, two monophyletic groups, (*Limnadopsis* + *Paralimnadia*) and (*Eulimnadia* + *Metalimnadia*), were recovered. *Imnadia* represented a sister group to ((*Limnadopsis*, *Paralimnadia*), (*Eulimnadia*, *Metalimnadia*)). *Limnadia* was the most basal group of the Family Limnadiidae.

#### 3.3.5. *28S* + *16S* + *EF1α* + *COI* Combined Analysis

**Figure 8:**
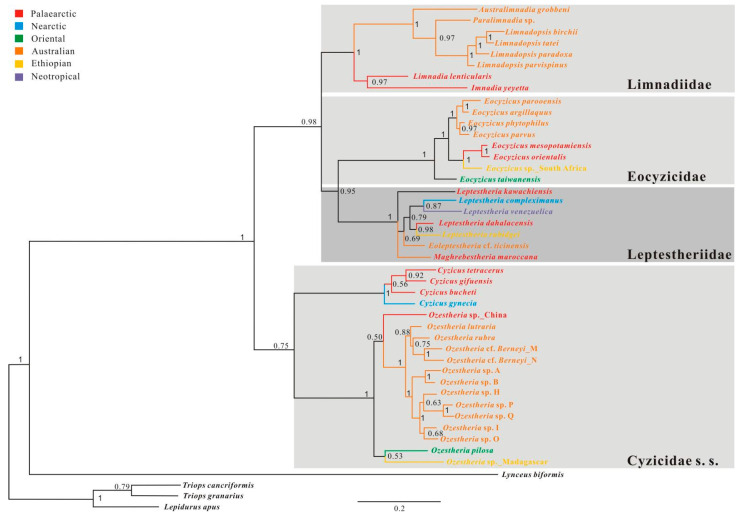
Bayesian tree of Spinicaudata (color-coded) derived from mitochondrial *16S rRNA* and *COI*, and nuclear *28S rRNA* and *EF1α* gene sequence data sets with Bayesian posterior probabilities (BPP) ≧0.75 reported above the nodes.

Appropriate and extensive taxon sampling has improved the accuracy of phylogenetic inferences of Spinicaudata [4]; while full four-gene sequences are still only available for about 53 species, large collections of partial gene sequences are available for about 111 species of Spinicauata. Thus, the alignment of partial gene sequences results in a gap-rich sequences alignment which is arranged in a staggered pattern [62], and the presence of numerous alignment gaps inevitably leads to the uncertainty of positional homologies, as observed in other rRNA studies [63]. To reduce the bias derived from a gappy multiple sequence alignment, we performed a phylogenetic analysis based on *28S + 16S + EF1α + COI* genes sequences. The taxa with the most strongly deviating composition were excluded from the analysis. Saturation and best models were estimated respectively for five sorts of subsets of the concatenated data set: the stem sites of rRNA genes, the loop sites of rRNA genes, the first codon positions, the second codon positions, and the third codon positions of protein-coding genes. As the third-codon positions of *EF1α* and *COI* genes showed significant nucleotide saturation, they were excluded from phylogenetic analyses. Substitution saturation test revealed no saturation for the concatenated sequences of *28S + 16S + EF1α + COI* genes after removing third codon positions, with the value of Iss significantly lower than the critical values (Iss.c) (Table 1: 0.614 < 0.807, *p* = 0.00). The Bayesian analysis of the combined data set of all four genes with structural partitions recovered 4 major monophyletic groups (Figure 8): Leptestheriidae (BPP = 1.0), Eocyzicidae (BPP = 1.0), Limnadiidae (BPP = 1.0), and Cyzicidae s. s. (BPP = 0.75). The key difference compared to other data sets analysis was that the phylogenetic relationships among Leptestheriidae, Eocyzicidae, and Limnadiidae were significantly resolved. A sister group relationship between Leptestheriidae and Eocyzicidae was recovered with high support (BPP = 0.95), and Limnadiidae was a sister group to Leptestheriidae + Eocyzicidae (BPP = 0.98). This branching pattern is congruent with a previous study [14] based on transcriptome data set analyses. Within the Family Limnadiidae, the resulting tree with high support values (BP = 1) revealed that monophyletic (*Imnadia* + *Limnadia*) (BP = 0.97) was a sister lineage to all other limnadiids. For Leptestheriidae, most nodes were resolved, including at nodes that have been difficult to resolve, such as the position of *Eoleptestheria*.

### 3.4. Testing Support for Competing Hypotheses

**Figure 9:**
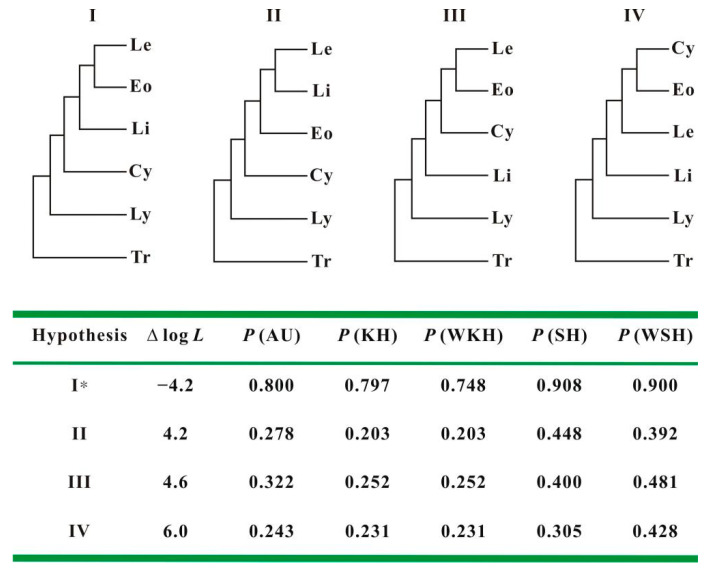
Statistical tests for three competing hypotheses. Statistical tests of significance were conducted for different competing phylogenetic hypotheses within spinicaudatan families, using combined data sets by the nonparametric bootstrapping tests, ranked by likelihood. In tree topologies, the abbreviations used are: Le, Leptestheriidae; Eo, Eocyzicidae; Li, Limnadiidae; Cy, Cyzicidae s. s.; Ly, Lynceidae; Tr, Triopsidae; AU: approximately unbiased test; KH: the Kishino–Hasegawa test; WKH: weighted Kishino Hasegawa test; SH: the Shimodaira–Hasegawa test; WSH: weighted Shimodaria Hasegawa test; *: the best tree.

The results of constrained nonparametric bootstrapping analyses of combined all four genes data set are summarized. To test the relative positioning of Leptestheriidae, Eocyzicidae, Cyzicidae s. s., and Limnadiidae in more detail, we first compared four alternative topologies. Results of the statistical tests indicated that the difference was not statistically significant (Figure 9), although the topology I came close to the significance threshold (AU = 0.800; KH = 0.798; SH = 0.908).

To test the relative positioning of *Eulimnadia*, *Imnadia*, and *Limnadia*, we compared four alternative topologies (Table 2). Topology 1 was designed to test the relationship proposed by previous studies [4,15,16]. According to this hypothesis, *Eulimnadia* would be more closely related to gondwanian limnadiids and (*Imnadia*, *Limnadia*) as the basal clade. The second topology was designed to test the relationships derived from an analysis of *28S rRNA* + *EF1α* (Figure 8), which supports a close relationship of *Imnadia* to (*Eulimnadia*, gondwanian limnadiids). The third topology was designed to test the relationship derived from the combined four-gene analyses conducted by Schwentner et al. [4]. Topology 4 was designed to test the relationship proposed by Hoeh et al. [12]. This hypothesis proposes *Imnadia* as a sister group to (*Eulimnadia*, Metalimnadia). The hypothesis of *Eulimnadia*, being the most basal group of Limnadiidae, was rejected (AU = 0.018), and topology 1 ((((*Limnadopsis*, *Paralimnadia*), *Australimnadia*), *Eulimnadia*), (*Imnadia*, *Limnadia*)) came close to the significance threshold (Table 2: AU = 0.676; KH = 0.651; SH = 0.818).

We also applied the same tests to the relationships within Leptestheriidae. Hypothesis 1 was designed to test the relationships derived from analyses of *28S rRNA* data (Figure 5) and *28S* + *16S rRNA* data (Figure 6), which supported a close relationship of *Leptestheria kawachiensis* to (*Maghrebestheria* + *Eoleptestheria*). Hypothesis II was designed to test the relationship derived from the combined four-gene analyses conducted by Schwentner et al. [4]. The hypothesis of *Maghrebestheria*, as the most basal group, was strongly rejected (Table 3) in favor of the proposed monophyly of ((*Maghrebestheria* + *Eoleptestheria*) + *Leptestheria kawachiensis*).

## 4. Discussion

### 4.1. Ribosomal DNA Polymorphism and Pseudogenes

The polymorphism of ribosomal DNA has been associated with the existence of pseudogenes in the genome. Pseudogenes are usually characterized by truncated sequences, more insertion/deletion sites, higher rates of base substitutions, lower GC content, a lower minimum free energy, a less stable secondary structure, and variation in the conserved regions [64]. Non-compensatory (CBCs) base changes that can disrupt the structure may be equally or more important criteria in identifying putative rRNA pseudogenes. Smith and Bond [65] found the helices G3–G15 to be the hypervariable regions which showed marked variations in domain V of *16S rRNA* in spiders and other arachnids; they also documented an overall evolutionary trend towards reduction in the hypervariable region in advanced arachnids. High variations in both length and secondary structure in E18, G3 and G9 helices of *16S rRNA* also occurred in most insects, with no apparent conserved motifs to aid in alignment [66]. Gillespie et al. [67] reported two structures for the braconids, illustrating the extreme variation in the Family Ichneumonoidea, and documented a large deletion event in the V2–C5 helix (helix 3-2) of *28S rRNA* in *Ephedrus* spp. Our comparative structure analysis also indicated that variations found in the *28S rRNA* and *16S rRNA* could be interpreted as a true phenomenon and not as pseudogenes due to higher GC content, high sequence similarity, mostly CBCs or SBCs (semi-compensatory) base changes to maintain the structure, and conserved motifs.

### 4.2. 28S rDNA Polymorphism and Mode of Reproduction

Spinicaudatans display a wide diversity of reproductive modes: dioecy (separate males and females), hermaphroditism and androdioecy (mixtures of males and hermaphrodites) [15,54,55,57,58,59,60,68,69]. The males were considered to play an important role in the population genetic diversity of branchiopods [70]. Crease and Lynch [21] suggested that cross-fertilization might increase the intraspecific variation of *Daphnia pulex* due to recombination. On the other hand, some studies also reported high rDNA diversity in asexual organisms [22,71]. A high level of *28S rDNA* polymorphism for some spinicaudatan species was recognized for the first time in the current study. We mapped the presence/absence of each type of V2-C5 structures and reproductive modes onto the combined-data phylogeny for spinicaudatans to analyse the correlation of rDNA polymorphism with reproductive modes (Figure 7). Due to the length heterogeneity in D2 segment (V2-C5 helix), two secondary structure types (type I and type II) were recovered among spinicaudatan species, especially within different populations of a single species. As displayed in Figure 7, the sequence variations of *28S* gene within dioecious species (*Leptestheria compleximanus, Limnadopsis parvispinus*, *L. taei*, *Paralimnadia stanleyana*, *P. badia*, *P. sordida* and *Imnadia yeyetta*) and androdioecious species (*Eulimnadia africana*) were considered to be high, suggesting that potential cross-fertilization might increase the offspring genetic diversity. The mode of reproduction in *Eulimnadia cylindrova* has been thought to be hermaphroditism or androdioecy [15,57,58,59]. Multiple specimens per species of *E. cylindrova* represented non-monophyletic in the phylogenetic analyses by a previous study [15], and all populations of this species were classified into an extended species group, *E. cylindrova* sensu lato [72]. The length heterogeneity in the *28S* V2-C5 helix was observed within androdioecious populations: type I occurred in Neotropical and Nearctic populations, and type II occurred in the Palaearctic population (Figure 7). In the case of three cryptic species of *Limnadia lenticularis*, both types were identified in hermaphroditic populations of *L. lenticularis* from the Palaearctic, while only type I occurred in hermaphroditic populations of *L. nipponica* from the Sino-Japanese region, and only type II in hermaphroditic populations of *L. americana* from the Neartic. *L. lenticularis* was composed of three cryptic species (*L. lenticularis*, *L. nipponica*, *L. americana*), with allopatric distribution in Europe, East Asia and America [73], which might suggest that the most recent speciation events of three *Limnadia lenticularis* cryptic species preceded the duplication event lacking concerted evolution. Our results also suggested that some spinicaudatan species might have undergone a relaxed concerted evolution with respect to *28S rRNA* gene. Further study, including additional populations of *Eulimnadia*, is needed to better understand the relationships between rDNA polymorphism and mode of reproduction.

### 4.3. Potential Phylogenetic Signal for Resolving Spinicaudatan Phylogeny

Non-phylogenetic signal misleads phylogenetic inference of ancient rapid divergences, resulting in weak support or strong support for artifactual topologies [74,75,76]. The comparison of different competing phylogenetic hypotheses derived from differently fitting models of sequence evolution and homoplasy testing with different data sets has been used to determine the congruence of the phylogenetic signal and to explore the effects of non-phylogenetic signal on phylogenetic reconstructions [77,78,79,80,81]. In this study, a comparison of different analyses indicated that major monophylogenetic groups were revealed within Spinicaudata with high supports. However, relationships among these groups still remain ambiguous, and different data sets support incompatible relationships (Figure 4, Figure 5, Figure 6, Figure 7 and Figure 8).

The tree derived from *28S rRNA* analysis favors Eocyzicidae as the closest relative to Leptestheriidae with high support, which was congruent with the conclusion of Schwentner et al. [11] (Hypothesis III in Figure 1b and Figure 9). Meanwhile, combined analysis of *28S* and *16S rRNA* suggested a closer relationship of Leptestheriidae with Limnadiidae (BPP = 0.56), which was congruent with the conclusion of Schwentner et al. [4] (Hypothesis II in Figure 1d and Figure 9). These two phylogenetic hypotheses were also incongruent with the inferences proposed by previous studies [12,13] (Hypothesis IV in Figure 1a and Figure 9). Our statistical tests indicated that these three hypotheses were not significantly different (Figure 9, AU values: 0.322 vs. 0.278 vs. 0.243). Although the doublet substitution model integration of secondary structures in alignment and tree reconstruction is an improved model superior to existing models for rRNA data set, limited phylogenetic signals have a major influence on the resulting topologies. The characteristics analysis of rRNA data set indicated that the current rRNA data sets contained only limited amounts of phylogenetic signal, due to the limited sequence length analyzed (Table 1), which was also the main source of these inconsistencies, because the small amount of phylogenetic signal could be easily swamped by a combination of systematic and stochastic error [77]. On the other hand, sequence heterogeneity, covariation patterns of paired sites, and excessive homoplasy in loop regions still could obscure the phylogenetic signal under the doublet secondary structure-specific substitution model. The substitution saturation test revealed that the stem positions in current data sets contained more reliable phylogenetic signals, compared to loop regions, while loop positions clearly experienced excessive homoplasy (Table 1), suggesting that the strongest support might come from the non-phylogenetic signal. Thus, limited amounts of phylogenetic signal and excessive homoplasy in unpaired positions may be two of the major reasons that the current rRNA data sets are unable to solve deep node relationships and retrieved spurious groups.

Improving the quality of primary alignments, minimizing compositional heterogeneity, detecting multiple substitutions and using the most realistic model of sequence evolution have previously been used to reduce the non-phylogenetic signal [18]. The tree derived from the combined analysis of *28S + 16S + EF1α + COI* genes using the best fitting models and minimizing compositional heterogeneity, indicated that there was a strong phylogenetic signal supporting Limnadiidae as a sister group to Leptestheriidae + Eocyzicidae (Figure 7, BPP = 0.98), in keeping with the findings of Schwentner et al. [14] (Hypothesis I in Figure 1c and Figure 9). Furthermore, this topology came close to the significance threshold (AU = 0.800; KH = 0.798; SH = 0.908). Hence, high posterior probabilities of BI tree and approximately unbiased probabilities for Hypothesis I indicate rather good resolution of it.

A monophyletic group of ((*Limnadopsis*, *Paralimnadia*), *Australimnadia*) was consistently supported by different data sets analyses, in line with previous studies [4,15,16]. The results indicated that there was a strong phylogenetic signal supporting the monophyly of gondwanian limnadiids. A sister group relationship between *Imnadia* and *Limnadia* was supported by inferences from *16S rRNA* data, or concatenated *28S + 16S* data, or concatenated *28S + 16S + EF1α + COI* data (BPP = 0.89, 0.98, 0.94). These clearly demonstrated that additional source of phylogenetic signal to the support for this clade, which also suggested that the high support did not come from non-phylogenetic signal. Furthermore, the data set rejected the hypothesis of Schwentner et al. [4], i.e., *Eulimnadia* being the most basal group of Limnadiidae (Table 2). The monophyly of (*Imnadia*, *Eulimnadia*) is not rejected by the statistical tests, yet this monophyly was represented in only 2.3% of the post-burnin posterior probability distribution. Topology 1 ((((*Limnadopsis*, *Paralimnadia*), *Australimnadia*), *Eulimnadia*), (*Imnadia*, *Limnadia*)) came close to the significance threshold (Table 2: AU = 0.676; KH = 0.651; SH = 0.818). The current analyses indicated that the phylogenetic signal of the current data set for such deep nodes was strong.

A closer relationship of *Leptestheria kawachiensis* to (*Maghrebestheria*, *Eoleptestheria*) than to other leptestherids was consistently supported by phylogenetic inferences from *28S rRNA* data, or concatenated *28S + 16S* data (Figure 5 and Figure 6: BPP = 0.75, 0.72). The support for such a deep node decreased in combined analysis, which also indicated conflicting signals in rRNA data sets. *Leptestheria compleximanus*, being the earliest leptestheriid lineage, received significant support using rRNA data sets (Figure 5 and Figure 6, BPP = 0.99–1.0). However, Bayesian inference tree based on concatenated *28S + 16S + EF1α + COI* data indicated *Maghrebestheria* or *Leptestheria* *kawachiensis* as the basal branching lineages (Figure 8). The phylogenetic relationships within Leptestheriidae were not fully resolved. These results suggested that this part of trees is difficult to resolve based on current data sets and there remains a need to further clarify the deep relationships within Leptestheriidae, with increased sampling of taxa and data.

## 5. Conclusions

In summary, we dissected the potentials for phylogenetic noise and signal using four molecular loci and extensive taxon sampling, covering the major spinicaudatan clades. Our results indicated high heterogeneity across species of Spinicaudata, and the existence of conserved motifs in all spinicaudatan species. Correcting the candidate data set for systematic errors, such as substitutional saturation, model misspecification and compositional heterogeneity biases recovered a topology congruent with inferences from the transcriptome data of Schwentner et al. [14], whereby Limnadiidae was placed as a sister group to Leptestheriidae and Eocyzicidae with high support. Our study highlighted the importance of phylogenetic signal dissection for future attempts to resolve deep phylogenetic relationships among the branchiopods. A comprehensive and robust phylogeny of Spinicaudata might be reached by increasing more taxa and more genes in the near future, which will provide a better understanding of the complex morphological innovation of the spinicaudatans.

## Figures and Tables

**Table 1:** Characteristics of the applied test data sets, including the results of alignment characteristics and the test for substitution saturation.

Taxon	Gene	Species	Length *	P	Iss	Iss.c	P	C	V	Pi	S
*Cyzicus*	*28S*	7	537	stem	0.411	0.777	0.00	333	27	11	16
				loop	1.915	0.679	0.00	149	14	7	7
	*16S*	9	545	stem	2.133	0.644	0.00	235	37	34	3
				loop	1.370	0.645	0.00	198	58	50	8
*Ozestheria*	*28S*	27	549	stem	1.074	0.778	0.00	322	48	36	12
				loop	1.021	0.595	0.04	144	26	9	17
	*16S*	24	570	stem	0.643	0.659	0.88	214	67	53	14
				loop	1.254	0.682	0.00	172	96	71	25
*Eocyzicus*	*28S*	12	531	stem	1.043	0.777	0.00	322	32	6	25
				loop	1.470	0.651	0.00	156	14	4	10
	*16S*	20	517	stem	0.634	0.644	0.92	234	35	28	7
				loop	0.730	0.631	0.38	191	56	40	16
*Leptestheria* +*Eoleptestheria* +*Maghrebestheria*	*28S*	12	523	stem	0.170	0.777	0.00	327	26	22	4
				loop	0.922	0.664	0.16	153	17	13	4
	*16S*	16	517	stem	0.484	0.657	0.05	190	81	41	40
				loop	0.930	0.665	0.04	162	56	40	16
*Paralimnadia*	*28S*	7	477	stem	0.089	0.783	0.00	291	23	6	17
				loop	1.869	0.603	0.00	145	10	4	6
	*16S*	2	460	stem	-	-	-	-	-	-	-
				loop	-	-	-	-	-	-	-
*Limnadopsis*	*28S*	6	469	stem	0.015	0.780	0.00	300	12	11	1
				loop	0.206	0.647	0.00	151	9	6	3
	*16S*	4	515	stem	0.328	0.779	0.00	209	12	3	9
				loop	1.445	0.777	0.00	185	20	4	16
*Eulimnadia*	*28S*	16	482	stem	0.891	0.773	0.50	281	34	24	10
				loop	2.401	0.585	0.00	148	17	15	2
	*16S*	4	530	stem	0.251	0.780	0.00	263	12	2	10
				loop	1.723	0.758	0.00	227	10	3	7
Spinicaudata	*28S* & *16S* & *EF1α* & *COI*	40	2092	Stem, loop, 1st&2nd	0.614	0.807	0.00	1202	845	652	186

Iss: estimated index of substitution saturation for the data set. Iss.c: critical values for the index of substitution saturation. Iss > Iss.c (*p* < 0.05) indicates saturation. *: Alignment length, P: partition, C: conserved sites, V: variable sites, Pi: parsimony informative sites, S: singleton sites, 1st: the first codon position, 2nd: the second codon position, -: Could not be calculated.

**Table 2:** Statistical tests of alternative phylogenetic hypotheses of Limnadiidae using combined data sets by the nonparametric bootstrapping tests.

Item	Hypothesis	∆ log *L*	*p* Values				
			AU	KH	WKH	SH	WSH
1	((((*Limnadopsis*, *Paralimnadia*), *Australimnadia*), *Eulimnadia*), (*Imnadia*, *Limnadia*))	−1.5	0.676	0.651	0.651	0.818	0.835
2	(((((*Limnadopsis*, *Paralimnadia*), *Australimnadia*), *Eulimnadia*), *Imnadia*), *Limnadia*)	1.5	0.436	0.349	0.349	0.691	0.667
4	(((((*Limnadopsis*, *Paralimnadia*), *Australimnadia*), *Limnadia), Imnadia)*, *Eulimnadia*)	3.6	0.335	0.317	0.317	0.457	0.570
3	((((*Limnadopsis*, *Paralimnadia*), *Australimnadia*), (*Imnadia*, *Eulimnadia*)), *Limnadia*)	10.5	0.018	0.044	0.044	0.087	0.109

**Table 3:** Statistical tests of alternative leptestherid phylogenetic hypotheses using combined data sets by the nonparametric bootstrapping tests.

Item	Hypothesis *	∆ log *L*	*p* Values				
			AU	KH	WKH	SH	WSH
1	(((((Eole, Magh), Lkaw), ((Ldah, Lcor), Lrub), ((Lbre, Lspm), Lnob)), Lven), Lcom)	−628.1	1.000	1.000	1.000	1.000	1.000
2	((((((((Ldah, Lcor), Lrub), ((Lbre, Lspm), Lnob)), Lven), Lcom), (Eole, Lspm)), Lkaw), Magh)	628.1	6 × 10^−6^ **	0.000	0.000	0.000	0.000

*: Eole = Eoleptestheria ticinensis; Lbre = Leptestheria brevirostris; Lcom = L. compleximanus; Lcor = L. cortieri; Ldah = L. dahalacensis; Lkaw = L. kawachiensis; Lnob = L. nobilis; Lrub = L. rubridgei; Lspm = Leptestheria sp. (Madagascar); Lven = L. venezuelica; Magh = Maghrebestheria maroccana. ** Rejected with *p* < 0.01.

## Data Availability

All gene sequence data are available from GenBank (http://www.ncbi.nlm.nih.gov, accessed on 25 October 2021).

## Supplementary Materials

The following are available online at https://www.mdpi.com/article/10.3390/genes12111705/s1, Figure S1: Bayesian phylogram inferred from 16S rRNA gene, using a 16-state doublet model. Only Bayesian posterior probabilities equivalent to 1 are shown. Table S1: List of taxa and their sequences used in the current study. Table S2. Statistical comparison of the compositional heterogeneity observed across the taxa.
