# Supplementary material for: Phylogenetic Signal Dissection of Heterogeneous 28S and 16S rRNA Genes in Spinicaudata (Branchiopoda, Diplostraca)
Source: Genes (Basel). 2021 Oct 27;12(11):1705. doi: 10.3390/genes12111705 (PMC8625258; doi:10.3390/genes12111705)
Supplement: Supplementary file 1 [file genes-12-01705-s001.zip › Figure S1.pdf]

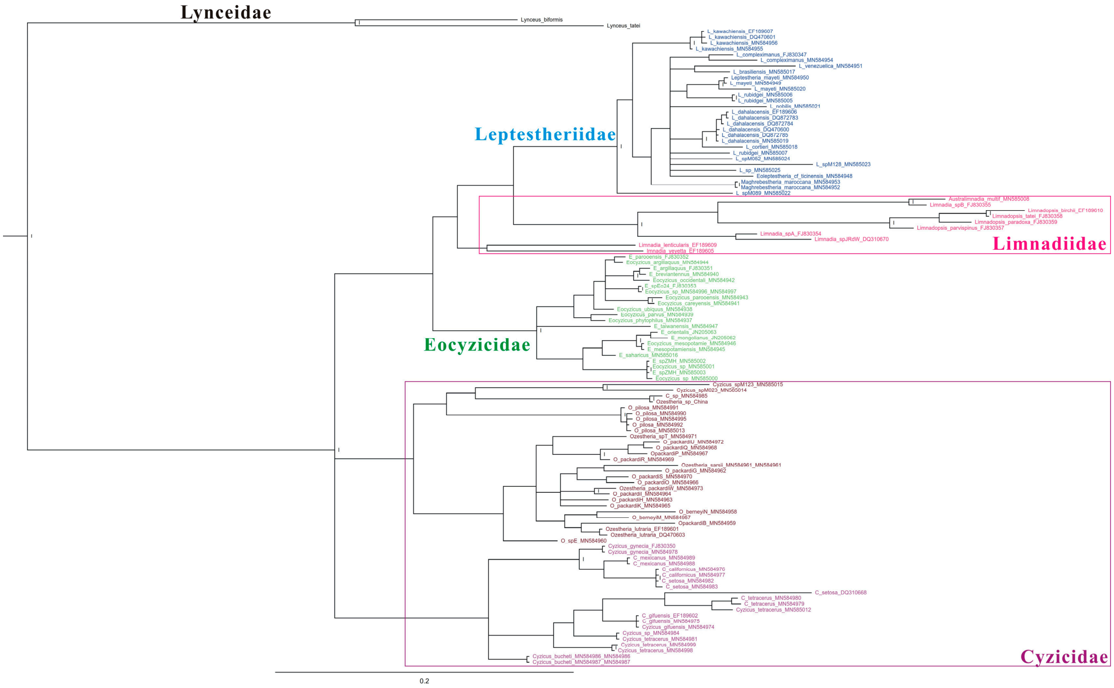

Figure S1. Bayesian phylogram inferred from 16S rRNA gene, using a 16-state doublet model. Only Bayesian posterior probabilities equivalent to 1 are shown.
