# Supplementary material for: Phylogenetic Signal Dissection of Heterogeneous 28S and 16S rRNA Genes in Spinicaudata (Branchiopoda, Diplostraca)
Source: Genes (Basel). 2021 Oct 27;12(11):1705. doi: 10.3390/genes12111705 (PMC8625258; doi:10.3390/genes12111705)
Supplement: Supplementary file 1 [file genes-12-01705-s001.zip › Table S2 Statistical comparison of the compositional heterogeneity.pdf]

Table S2. Statistical comparison of the compositional heterogeneity observed across the taxa.

	28S rRNA		16S rRNA	
Taxa	p-value	z-score	p-value	z-score
Cyzicus	0.6995	-0.5296	0.5165	-0.1420
Ozestheria	0.7340	-0.6916	0.4497	0.1537
Eocyzicus	0.5881	-0.5246	0.5224	-0.2174
Leptestheria	0.1860	0.8570	0.5976	-0.2980
Eoleptestheria	0.7750	-0.7090	0.2500	0.6930
Maghrebestheria	0.1160	1.0080	0.7495	-0.7145
Limnadia	0.7995	-0.7738	0.2000	0.5370
Limnadopsis	0.5085	-0.2336	0.5308	-0.3515
Imnadia	0.8435	-0.9958	0.3250	0.3950
Paralimnadia	0.4063	0.1551	0.2745	0.4705
Metalimnadia	0.4125	0.0825		
Eulimnadia	0.7659	-0.7329	0.04*	2.998*
Calalimnadia	0.5500	-0.3470		
Australimnadia	0.2320	0.8620	0.574	-0.296
Gondwanalimnadia	0.8750	-0.9160		

P-values indicate the statistical significance of test and z-scores quantify the amount of compositional heterogeneity (with * indicating significant values). A Z-score value of more than 2 ($p<0.05$) indicated that the taxa were significantly compositionally heterogeneous. The test statistic for the individual taxa indicated that the nucleotide composition of 8 specimens is significantly biased: *Leptestheria* sp. of India (Z=2.817, p=0), *Leptestheria brevirostris* (Z=4.763, p=0), *Ozestheria pilosa* (MN584994: Z=2.151, p=0.05; MN584993: Z=2.228, p=0.025), *Eulimnadia braueriana* (Z=3.013, p=0.025), *Eulimnadia* sp. C (Z=3.312, p=0.025), *Eulimnadia* sp. H (Z=2.156, p=0.025), *Eulimnadia* sp. K (Z=3.211, p=0.025). These seven taxa with the most strongly deviating nucleotide composition were excluded.
